# Human cells lacking coilin and Cajal bodies are proficient in telomerase assembly, trafficking and telomere maintenance

**DOI:** 10.1093/nar/gku1277

**Published:** 2014-12-03

**Authors:** Yanlian Chen, Zhiqiang Deng, Shuai Jiang, Qian Hu, Haiying Liu, Zhou Songyang, Wenbin Ma, Shi Chen, Yong Zhao

**Affiliations:** 1 Key Laboratory of Gene Engineering of the Ministry of Education, Cooperative Innovation Center for High Performance Computing, School of Life Sciences, Sun Yat-sen University, Guangzhou 510006, P.R. China; 2 Key Laboratory of Combinatorial Biosynthesis and Drug Discovery, Ministry of Education, School of Pharmaceutical Sciences, Wuhan University, Wuhan 430071, P.R. China

## Abstract

The RNA component of human telomerase (hTR) localizes to Cajal bodies, and it has been proposed that Cajal bodies play a role in the assembly of telomerase holoenzyme and telomerase trafficking. Here, the role of Cajal bodies was examined in Human cells deficient of coilin (i.e. coilin-knockout (KO) cells), in which no Cajal bodies are detected. In coilin-KO cells, a normal level of telomerase activity is detected and interactions between core factors of holoenzyme are preserved, indicating that telomerase assembly occurs in the absence of Cajal bodies. Moreover, dispersed hTR aggregates and forms foci specifically during S and G2 phase in coilin-KO cells. Colocalization of these hTR foci with telomeres implies proper telomerase trafficking, independent of Cajal bodies. Therefore, telomerase adds similar numbers of TTAGGG repeats to telomeres in coilin-KO and controls cells. Overexpression of TPP1-OB-fold blocks cell cycle-dependent formation of hTR foci and inhibits telomere extension. These findings suggest that telomerase assembly, trafficking and extension occur with normal efficiency in Cajal bodies deficient human cells. Thus, Cajal bodies, as such, are not essential in these processes, although it remains possible that non-coilin components of Cajal bodies and/or telomere binding proteins (e.g. TPP1) do play roles in telomerase biogenesis and telomere homeostasis.

## INTRODUCTION

Telomeres protect the termini of linear chromosome from degradation, end-to-end fusion and recombination (1). In human cells, the telomere is an ∼5–15-kb terminal chromosome region, whose DNA component includes tandem repeats of the motif 5′-TTAGGG/AATCCC-3′, and whose protein components include a telomere-specific complex called ‘shelterin’ (2). The terminal segment of linear eukaryotic chromosomes cannot be duplicated by traditional semi-conservative DNA synthesis; as a consequence, chromosomes in proliferating human somatic cell shorten by 50–300 bp per cell cycle, eventually triggering replicative senescence or apoptosis (3). Telomere shortening is not generally observed in human cancer cells, because the large majority of human cancer cells express active telomerase, a ribonucleoprotein with reverse transcriptase activity that adds telomeric DNA repeats to the end of telomeres in an RNA template-dependent manner (4,5). The telomerase ribonucleoprotein complex includes hTERT, the protein catalytic subunit (6); hTR, the catalytic telomerase RNA subunit (7); and dyskerin, a protein that stabilizes hTR (8). Assembled telomerase holoenzyme is transported or recruited to telomeres, by a mechanism that is not fully understood.

Cajal bodies are conserved subnuclear structures found in most eukaryotic cells, highly enriched in transcription factors, fibrillarin (Fb), survival motor neuron (SMN) protein complex and an 80-kDa structural protein, coilin (9,10). Small nuclear RNAs (snRNAs) and snRNPs are also enriched in Cajal bodies, where they are thought to undergo modification, maturation, splicing and/or assembly (11). Coilin acts as a scaffold for assembly of Cajal bodies and is thought to be essential for these processes. Mice carrying a homozygous null allele of the gene encoding coilin display reduced viability, fertility and fecundity, demonstrating that coilin is essential at the level of the organism (12). A motif matching the consensus sequence of H/ACA box acts as a localization/targeting sequence, identifying small RNA molecules for transport to Cajal bodies (13). This motif, called a CAB box, is present in the 3′ stem-loop of telomerase catalytic RNA, hTR (14), which aggregates into ‘hTR foci’ associated with hTERT in Cajal bodies (15,16). In proliferating human cells, Cajal bodies co-localize with replicating telomeres during S phase (14), and TCAB1 (telomerase Cajal body protein 1), an essential subunit of Cajal bodies, interacts with active telomerase enzyme and is responsible for telomerase localized to Cajal bodies (17).

It has been proposed that Cajal bodies may serve as sites for telomerase maturation, assembly and function to deliver telomerase holoenzyme to telomeres during S phase; however, some discrepancies exist. For example: (i) mouse telomerase RNA (mTR) does not localize to Cajal bodies, but it does form foci on a subset of telomeres during S phase (18); (ii) telomerase is able to be recruited to telomeres in a Cajal body-independent manner in human cancer cells (19). Moreover, it has been found that in fruit flies lacking Cajal bodies due to a knockout mutation in coilin, resident small Cajal bodies RNAs (scaRNAs) are modified and function normally (20). The significance of these discrepancies remains unknown, and it remains unclear whether and how Cajal bodies promote telomerase biogenesis and/or telomere maintenance in human cells.

The goal of this study was to directly test whether Cajal bodies are required for telomerase biogenesis and function in human cells. For this purpose, a coilin null allele was introduced into HeLa cells using targeted zinc-finger nuclease (ZFN)-mediated insertional mutagenesis, resulting in a coilin-knockout (coilin-KO) cell line in which Cajal bodies were not detected. Coilin-KO HeLa cells lack constitutive hTR and TCAB1 foci, but hTR/TCAB1 foci are detected transiently at a subset of telomeres during S phase. Telomerase activity and function appear to be normal in coilin-KO HeLa cells because (i) telomere attrition was not observed *in vivo*; (ii) TTAGGG repeat addition by telomerase was efficient; and (iii) telomerase holoenzyme assembly proceeded similarly in coilin-KO and wild-type cells. Furthermore, overexpression of TPP1 OB-fold blocked the formation of hTR/TCAB1 foci in coilin-KO HeLa cells during S phase, leading to telomere attrition. The implications of the findings are discussed.

## MATERIALS AND METHODS

### Cell culture

HeLa, HTC75 and 293T cells were cultured at 37ºC under 5% CO_2_ in Dulbecco's modified Eagles’ medium (DMEM) supplemented with 10% new calf serum (HyClone) and 100U/ml penicillin and streptomycin (HyClone). siRNAs of coilin (Invitrogen) were transfected into HTC75 and 293T cells with Lipofectamine 3000 following the instruction provided by manufactory (Invitrogen).

### Knockout of coilin in HeLa cells using ZFN system

ZFN coilin R and ZFN coilin F were purchased from Sigma-Aldrich. 2 × 10^5^ HeLa cells in 6-well plates were co-transfected with 3μg ZFN *coilin* R and 3μg ZFN *coilin* F using Lipofectamine 2000. Transfected cells were grown on solid media and screened for coilin-KO using polymerase chain reaction (PCR). Seventeen of 214 clones analyzed by PCR carried a heterozygous insertion mutation at coilin. The transfection protocol was repeated, with the heterozygous cells, and transfectants were screened for homozygosity for the null allele at the coilin locus.

### Cell cycle synchronization

Exponentially growing HeLa cells were synchronized in DMEM plus 2mM thymidine for 19 h, washed 3× with PBS, transferred to and incubated in fresh pre-warmed DMEM for 10 h, then DMEM plus 2mM thymidine for 14 h, washed 3× with PBS, and finally transferred to DMEM and incubated for 0–10 h.

### Fluorescence *in situ* hybridization and immunofluorescence analysis of hTR TCAB1 and components of Cajal bodies

Fluorescence *in situ* hybridization (FISH) was performed as described (21). As a control, cells were spread on a slide for hybridization and pre-treated with 0.2mg/ml RNase A at 37°C for 2 h before the FISH hybridization probe was added. Fluorescein isothiocyanate-conjugated DNA probes complementary to human telomerase RNA were synthesized by TAKARA Bio Inc. Coilin and/or TCAB1 were detected by immunofluorescence (IF). Antibodies to coilin and TCAB1 were from Abcam (UK). Cy5-conjugated secondary antibody (Multiscience, China) was used for detection. Cells were mounted and images were acquired with a Zeiss Axio Imager Z1 Microscope equipped with an AxioCamMR3 camera using 40× or 63× oil immersion lenses. Images were processed using AxioVision Rel. 4.6.

### TRAP assay

TRAP (Telomeric Repeat Amplification Protocol) telomerase activity assay was performed as described (22). Relative telomerase activity was calculated as a ratio of signal in TRAP ladder to signal of an internal control.

### Telomere Restriction Fragment assay

Genomic DNA was isolated from cultured cells using AxyPrep Blood Genomic DNA Miniprep kit (Axygen). Telomere length was estimated as previously described (23).

### Fluorescence-activated cell sorting

Cells were released from monolayers by trypsinization and fixed in 70% ethanol overnight at 4°C. Cells were stained with propidium iodide (25μg/ml) in solution containing RNase A (100μg/ml). Cells were sorted and relative fluorescent signal was measured using FACScan (Becton Dickinson). Data were analyzed using Cell Quest software (Becton Dickinson).

### In gel hybridization assay to determine overhang length

In gel hybridization assay was performed as previously described (24).

### dsDNA-specific nuclease overhang assay

dsDNA-specific nuclease (DSN) assay was performed as described (25).

### Biomolecular fluorescent complementation assay

Biomolecular fluorescent complementation (BiFC) assay was performed as previous described (26). Briefly, DKC1 gene was cloned into pBabe-CMV-CYFPn-DEST-neo vector, hTERT and TCAB1 were cloned into pCL-CMV-CYFPc-DEST-puro vectors. Lentiviral particles carrying DKC1-YFPn or hTERT-YFPc or TCAB1-YFPc were produced by co-transfecting to HEK293T cells with expression plasmid(s), pCGP and VSVG, as appropriate. The supernatant was collected and used to infect target cells (Clone 12, 44 and control HeLa cells). Cells stably expressing DKC1-YFPn were isolated, and then re-transfecting as appropriate (i.e. hTERT-YFPc or TCAB1-YFPc).

### Overexpression of coilin or TPP1 OB-fold in HeLa cells

Retroviral particles were generated by co-transfecting 293T cells with packaging plasmids pCGP and pVSVG and expression vectors encoding HA-FLAG-tagged coilin or HA-FLAG-tagged TPP1 OB-fold. Viral supernatants were collected after 48 h and used to infect target cells. Infected cells were selected by growth in the presence of puromycin (2 μg/ml) for 7 days.

### IP (Immunoprecipitation) -TRAP

Anti-Flag immunoprecipitates from TCAB1-expressing HeLa cells were eluted with FLAG peptides (Sigma), diluted and used for q-TRAP assay. Q-TRAP was carried out essentially as described (27). Each 25μl Q-TRAP reaction contained 2μl FLAG-eluted immunoprecipitates, 100ng TS primer (5′-AATCCGTCGAGCAGAGTT-3′), 100ng ACX primer (5′-GCGCGGCTTACCCTTACCCTTACCCTAACC-3′) and 1mM EGTA (Ethylene Glycol Tetraacetic Acid) in SYBR Green PCR Master Mix (Applied Biosystems). The reaction mixtures were incubated at 30°C for 30 min and then PCR-amplified for 40 cycles at 95°C for 15 s and at 60°C for 60 s using an ABI StepOnePlus real-time PCR system (Applied Biosystems).

## RESULTS

### Mutagenesis of coilin gene in HeLa cells

**Figure 1.**
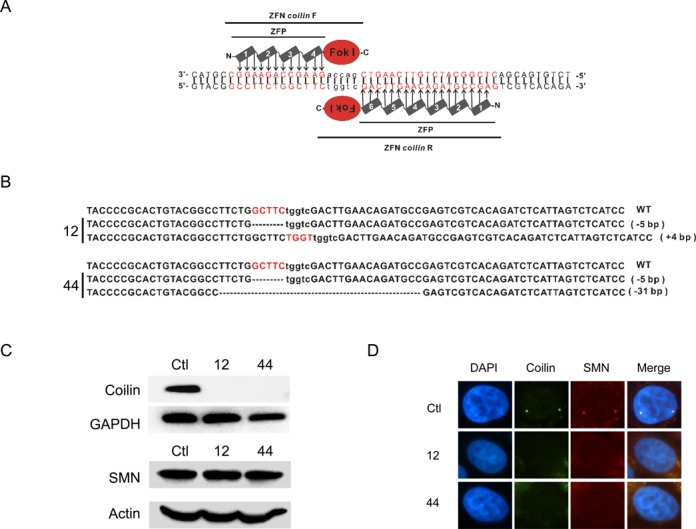
Insertional mutagenesis of coilin in HeLa cells. (**A**) Schema for insertional mutagenesis using ZFN system. (**B**) DNA sequence of null coilin allele in clones 12 and 44. Mutant alleles are shown aligned with wild-type coilin. (**C**) Western blot of protein extracts of HeLa cells or coilin-KO HeLa cells 12 or 44. Blots were probed with antibody to coilin, SMN, actin or GADPH (control). (**D**) IF (Coilin and SMN) to detect the presence of Cajal bodies in HeLa and coilin-KO cells.

Coilin acts as a scaffold during assembly of Cajal bodies (28) and previous studies in insect cells show that a null allele of coilin (i.e. coilin-KO) blocks formation of Cajal bodies (29). Mouse embryonic fibroblasts lacking functional coilin also fail to form Cajal bodies (30). To extend these studies to a human cell-based system, ZFN technology was used to generate a KO allele of coilin. Two ZFN (ZFN coilin F and ZFN coilin R) was designed, each consisting of Fok I nuclease at C-terminal and zinc-finger protein at N-terminal that specifically recognizes and binds to target sequence (Figure 1A). The sequence ranging from 72 to 109 downstream of the coilin start codon (ATG) was selected as a target. Two Fok I nucleases formed heterodimer that produced a double-stranded break (DSB) on target sequence (Figure 1A). Imperfect repair of DSB provides the possibility for genome editing. Mutagenesis of coilin gene was introduced into HeLa cells in a two-step transfection experiment. Two clones, 12 and 44, were selected for characterization, both of which carry the null alleles of coilin.

DNA sequencing, western and functional studies confirm that clones 12 and 44 fail to express functional coilin (Figure 1B–D). A previous report indicated that cells depleted for coilin by siRNA knockdown grow more slowly than control cells (31); in contrast, the coilin-KO HeLa cells used in this study grow at the same rate as coilin-expressing control cells (data not shown). SMN protein, an intrinsic component of Cajal bodies, was used to characterize the presence of Cajal bodies. Western blots show that coilin knockout resulted in no change of SMN expression (Figure 1C). In control cells, SMN foci colocalized with coilin, as expected. In coilin-KO cells, we observed complete loss of SMN foci, indicating the absence of Cajal bodies (Figure 1D).

### Effect of coilin-KO on hTR foci, TCAB1 foci, telomerase activity and telomere length

**Figure 2.**
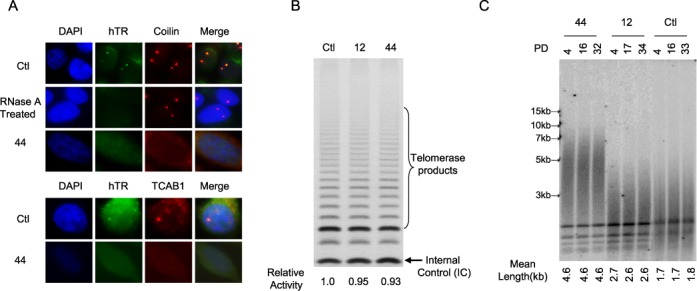
hTR foci and telomerase activity in coilin-KO cells. (**A**) RNA-FISH (hTR) and IF (TCAB1 and coilin) in HeLa and coilin-KO cells. (**B**) TRAP assay in HeLa and coilin-KO cells. (**C**) TRF assay over 30 population doublings (PDs) in HeLa and coilin-KO cells.

Initial characterization of coilin-KO HeLa cells also showed absence of hTR and TCAB1 foci (detected by three-probe FISH and IF, respectively) (Figure 2A), despite normal expression levels of hTR and TCAB1 (data not shown). These results confirm that coilin is required for the formation of Cajal bodies, and raise the possibility that hTR and TCAB1 are likely dispersed in the nucleoplasm in the absence of Cajal bodies.

*In vitro* TRAP assay was used to measure relative abundance of telomerase in extracts from coilin-KO and control cells. The results indicate that levels of telomerase activity are unaffected by absence of coilin and Cajal bodies (Figure 2B), suggesting that neither coilin nor Cajal bodies are required for maturation of hTR and assembly of telomerase holoenzyme. The effect of Cajal bodies on telomerase activity was further tested in human 293T and HTC75 cells. The depletion of coilin by siRNAs led to a lack of detectable Cajal bodies and TCAB1 foci, but had no effect on telomerase activity (Supplementary Figure S1). Consistent with this conclusion, direct measurement of telomere length using the Telomere Restriction Fragment (TRF) assay shows no evidence of telomere shortening in coilin-KO cells for >30 population doublings (PDs) (Figure 2C).

**Figure 3.**
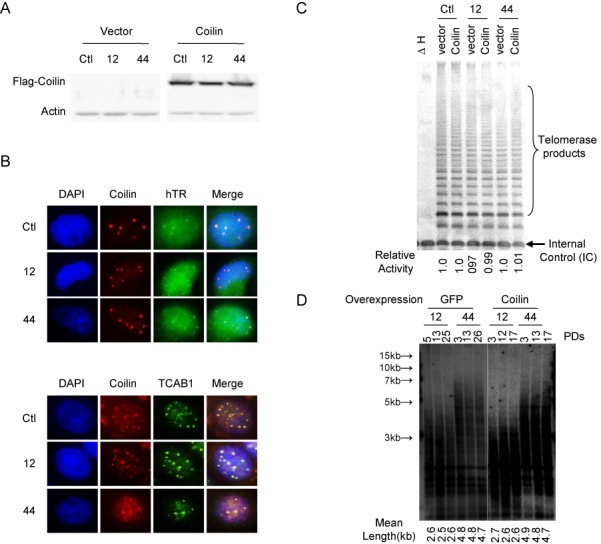
Genetic complementation of coilin-KO HeLa cells. (**A**) Western blot of protein extracts from cells overexpressing FLAG-tagged coilin or FLAG epitope only (i.e. vector control) using antibody to FLAG epitope. Cell lines used are HeLa (Ctl) and coilin-KO 12 and 44. (**B**) FISH and IF of cells used in the right panel of (A). (**C**) As in Figure 2B, using cell lines analyzed in (A). (**D**) As in Figure 2C, using cell lines analyzed in (A). The number of population doublings is indicated at top of each gel lane.

To confirm that ZFN-mediated insertion selectively inactivated coilin and that the phenotype of coilin-KO cells reflects only this mutation event, rescue experiment was performed in which DNA encoding FLAG-tagged coilin was introduced into the cells via viral particle vectors. The infected cells expressed in high levels of FLAG-tagged coilin (Figure 3A), which rescued/complemented the defect in formation of Cajal bodies (Figure 3B). hTR and TCAB1 foci reoccurred and also colocalized with Cajal bodies in complemented coilin-KO cells (Figure 3B). Telomerase activity was identical (Figure 3C), and telomere length largely maintained over PDs in wild-type control and complemented coilin-KO cells (Figure 3D and Supplementary Figure S2).

### Assembly of telomerase holoenzyme in coilin-KO cells

**Figure 4.**
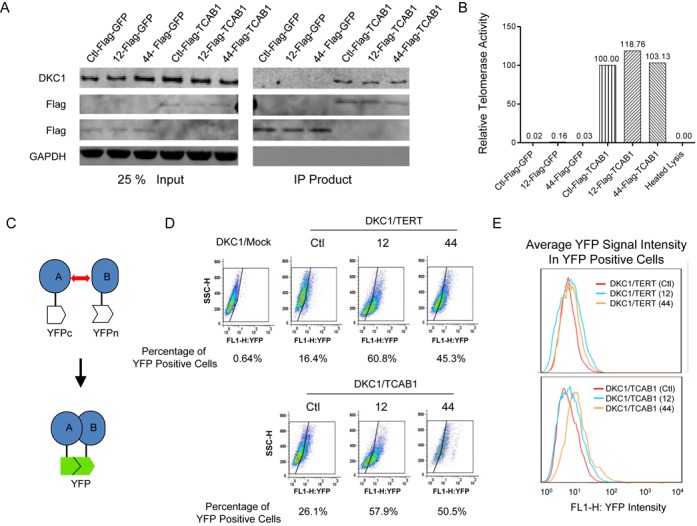
Telomerase assembly in coilin-KO cells. (**A**) IP of TCAB1 analyzed by western blot with antibody to DKC1. Cell lines are indicated. (**B**) Telomerase pulled down by antibody against FLAG-TCAB1 was assayed by q-TRAP. (**C**) Schematic diagram showing fusion proteins used in BiFC experiment. Protein A is DKC1; Protein B is hTERT or TCAB1, as indicated in panels (**D**) and (**E**). (D) FACS analysis in HeLa or coilin-KO HeLa cells co-expressing BiFC variants of DKC1, TERT and TCAB1 as indicated. Cells were sorted by relative YFP fluorescence. (E) Quantitative analysis of YFP fluorescence intensity from panel (D) represented as histograph.

Telomerase holoenzyme includes hTERT, hTR, DKC1 and TCAB1. DKC1 stabilizes hTR (8), and TCAB1 is thought to facilitate holoenzyme subunit interactions as well as its recruitment to Cajal bodies and telomeres (32). In addition, TCAB1 is a major component of Cajal bodies. To examine the effect of coilin-KO and the absence of Cajal bodies on telomerase subunit interactions, FLAG-tagged TCAB1 was expressed in wild-type control (Ctl) and coilin-KO cells (12 and 44), and antibody to the FLAG epitope was used to co-immunoprecipitate TCAB1-containing complexes and test them for functional telomerase holoenzyme. As an additional control for specificity, FLAG-tagged GFP (Green Fluorescent Protein) was expressed in the cell lines instead of FLAG-tagged TCAB1. The results show that extracts of control and coilin-KO cells form complexes containing DKC1 and TCAB1 with similar efficiency (Figure 4A) and that the immunoprecipitated complexes contain a comparable level of hTR (Supplementary Figure S3A) and telomerase activity (Figure 4B and Supplementary Figure S3B). Therefore, we conclude that functional telomerase holoenzyme is assembled efficiently in coilin-KO cells that lack Cajal bodies.

BiFC assay was also used to investigate telomerase holoenzyme assembly in coilin-KO cells. BiFC resembles a two-hybrid interaction screen, in which fluorescence output is a measure of the proximity of N- and C-terminal fragments of Yellow Fluorescent Protein (YFP). Here, the N-terminal fragment of YFP was expressed as a fusion protein derivative of DKC1, and the C-terminal fragment of YFP was expressed as fusion protein derivatives of hTERT or TCAB1 (Figure 4C). Pairs of N- and C-fusion proteins were co-expressed in control and coilin-KO cells, and then proliferating cells were analyzed by fluorescence-activated cell sorting (FACS). The results show that the percentage of YFP-positive cells and the average YFP signal are higher in coilin-KO than in wild-type control cells (Figure 4D and E). This indicates that in our experimental conditions, telomerase holoenzyme assembly is more efficient in coilin-KO cells than it is in control cells. A possible explanation is that in the absence of Cajal bodies, hTR is dispersed (Figure 2A) and the assembly of telomerase holoenzyme could occur everywhere in nucleoplasm. This is more efficient than that all components are delivered to Cajal bodies for assembly.

### Effect of coilin-KO on extension of telomeric overhangs during S phase

**Figure 5.**
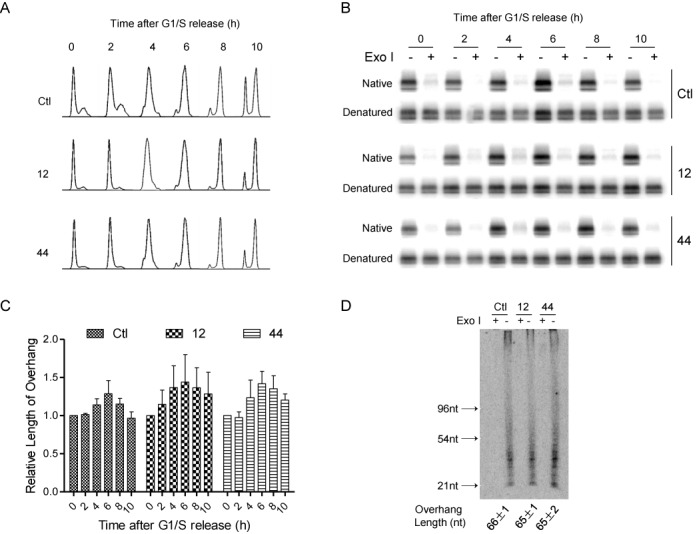
Quantifying telomere overhang length in HeLa and coilin-KO HeLa cells. (**A**) HeLa and coilin-KO cells were synchronized at G1/S, released into S and analyzed by FACS at the indicated time points. Cells were sorted according to DNA content. (**B**) As in (A), except that cells were harvested, genomic DNA isolated, cleaved by restriction enzyme and analyzed by agarose gel under native and denatured conditions. DNA was blotted and probed with telomere-specific probe. (**C**) Quantitation of data in (B), three independent experiments were performed. Mean overhang length ± SD was calculated. (**D**) DSN assay was used to estimate average overhang length in wild-type and coilin-KO cells.

Under homeostatic conditions that prevent telomere shortening in proliferating cells, telomerase adds ∼60nt *de novo* TTAGGG repeats to the 3′ overhang of all (or most) telomeric chromosome ends during S phase. Synthesis of the complementary C-rich DNA strand occurs during late S/G2, such that 3′ overhangs appear transiently longer during S phase than during late S/G2 (23). Therefore, transient lengthening of 3′ overhangs during S phase was evaluated in control and coilin-KO cells, as an estimate of telomerase extension activity *in vivo* on telomere ends (33). For this purpose, cells were synchronized at G1, released to enter S phase, and genomic DNA was isolated at 2 h intervals. Purified genomic DNA was assayed for determining relative overhang length by Southern blot under native condition. The rate of cell cycle progression was identical in control and coilin-KO cells (Figure 5A) so that relative overhang length could be compared at each time point (Figure 5B). Quantitative analysis of the data demonstrates that average overhang length peaks 6 h after release from G1/S in control and coilin-KO cells (Figure 5C) and there is no significant difference in relative overhang length in wild-type and coilin-KO cells during S phase. Because these data compare relative overhang length, genomic DNA was also treated with DSN, and 3′ telomeric overhang length was measured directly in control and coilin-KO cells (Figure 5D). These data confirm that average telomeric overhang length is not significantly different in control and coilin-KO cells during S phase. Slightly longer overhang at G2 (10 h after release) may be due to the delayed C-rich Fill-in (34).

### Telomerase trafficking in coilin-KO cells

**Figure 6.**
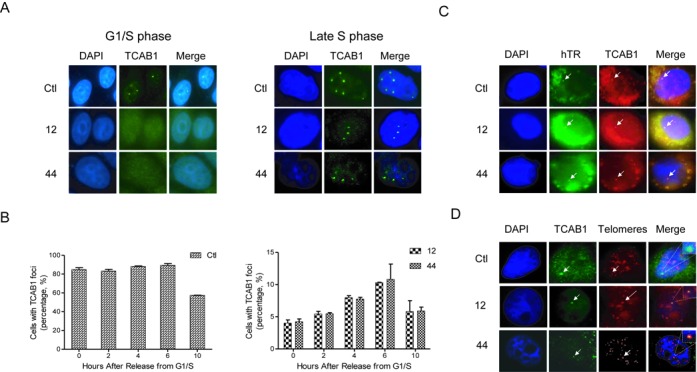
TCAB1/hTR foci and colocalization with telomeres at early and late S phases. (**A**) TCAB1 foci were analyzed as in Figure 2A, using cells at G1/S or late S phase, as indicated. (**B**) Cells were analyzed as in (A) at 2-h intervals after release from G1/S. Left panel: HeLa cells; right panel: coilin-KO cells. (**C**) FISH and IF analysis of TCAB1 foci and hTR during late S phase in the indicated cell lines. (**D**) FISH and IF analysis of TCAB1 foci and telomeric repeats during late S phase in the indicated cell lines.

It has been proposed that Cajal bodies facilitate telomerase trafficking and recruitment of multiple telomerase molecules to telomeres (21,35) and that the colocalization of hTR foci in Cajal bodies with telomeres represents an important step in this process (23). Because TCAB1 is a component of telomerase that associates with hTR, hTERT and DKC1, we chose TCAB1 as a tracer to detect the movement of telomerase. TCAB1 foci were tracked from G1/S through S phase in synchronized control and coilin-KO cells. Although TCAB1 foci are barely detected in asynchronous coilin-KO cells, they were clearly detected in synchronized cells starting in early S phase and peaking ∼6 h after release from G1/S (Figure 6A and B). hTR and TCAB1 followed a similar profile (Figure 6C) and ∼30% of the foci colocalized with telomeres (Figure 6D and Supplementary Figure S4), indicating that telomerase is able to be recruited to telomeres in the absence of Cajal Bodies.

### Role of TPP1 in the recruitment of telomerase to the telomere

**Figure 7.**
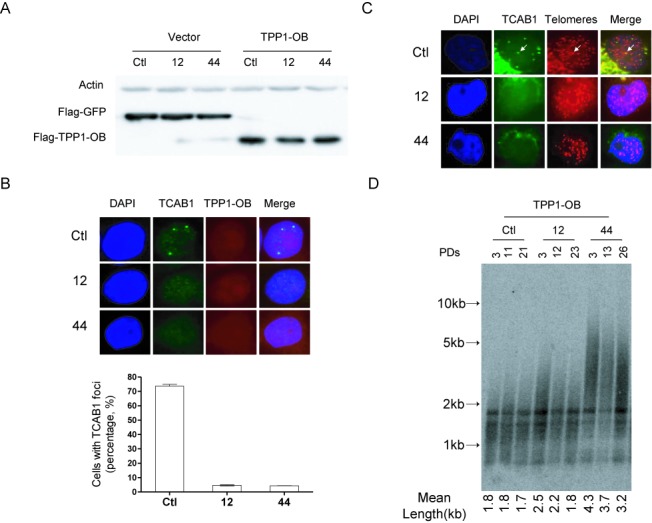
Overexpression of FLAG-tagged TPP1 OB-fold in coilin-KO cells. (**A**) Overexpression of FLAG-tagged TPP1 in wild-type and coilin-KO cells. Vector control is shown in the left panel. (**B**) TCAB1 foci and FLAG-tagged TPP1 OB-fold were detected by IF during late S phase in the indicated cell lines. Three independent experiments were performed; average values were calculated (graph below fluorescence images in the upper panel). (**C**) TCAB1 foci and telomere repeats were detected by IF or FISH in the indicated cell lines overexpressing FLAG-tagged TPP1 OB-fold. (**D**) Telomere length was assessed (as in Figure 2C) over the indicated number of population doublings in the indicated cell lines overexpressing FLAG-tagged TPP1 OB-fold.

Previous studies suggest that hTERT interacts specifically with the OB-fold of TPP1 at the telomere and that this interaction recruits telomerase to the telomere (36,37). Consistent with this, overexpression of the OB-fold of TPP1 in proliferating human cells interferes with telomere homeostasis, leading to telomere shortening (36). When the FLAG-tagged OB-fold of TPP1 was overexpressed in coilin-KO cells, the number of TCAB1 foci during late S/G2 (6 h after release from G1/S) decreased from 10.4% to 4.5% (clone 12) and from 10.8% to 4.3% (clone 44) (Figures 6B and 7B), while the number of TCAB1 foci in wild-type cells decreased only slightly (from 85% to 74%). This result suggests that TPP1 is implicated in the formation of TCAB1 foci when Cajal bodies are absent. Accordingly, no TCAB1 foci colocalizing with telomeres were observed in coilin-KO cells (Figure 7C). Furthermore, telomere length significantly decreases over time in proliferating coilin-KO cells (Figure 7D). These data are consistent with the idea that TPP1 plays a critical role in recruiting telomerase to the telomere and this role is conserved in human cells that lack coilin and Cajal bodies.

## DISCUSSION

This study tests the hypothesis that coilin and Cajal bodies play an essential role in telomere biology in human cells. Surprisingly, we show that coilin-KO human cells that lack Cajal bodies are phenotypically normal with respect to telomere homeostasis. In particular, (i) telomere length is maintained in coilin-KO HeLa cells; (ii) telomerase holoenzyme assembly proceeds normally in coilin-KO cells that lack Cajal bodies; (iii) hTR/TCAB1 foci are detected transiently during S phase at telomeres in coilin-KO HeLa cells; and (iv) active telomerase holoenzyme adds similar numbers of TTAGGG repeats to telomeres during S phase in coilin-KO and control HeLa cells. These data strongly refute the idea that Cajal bodies *per se* play an essential role in telomerase maturation, assembly or trafficking.

It is believed that Cajal bodies host RNA for modification and assembly reactions. However, this raises the question: do reactions that normally take place in a Cajal body also occur in the absence of morphologically recognizable Cajal bodies? Both wild-type and coilin-KO *Drosophila* are proficient in methylation and pseudouridylation of U1, U2, U4 and U5 snRNAs, suggesting that Cajal bodies may not be required for normal maturation of scaRNAs (20). This conclusion is strengthened by data presented here, which suggests Cajal bodies are dispensable for hTR maturation and assembly of active telomerase holoenzyme. While these functions do not require formation of Cajal bodies *per se*, it remains possible that non-coilin components of Cajal bodies do play specific roles in telomerase maturation, biogenesis, function and/or trafficking *in vivo*. Indeed, the depletion of TCAB1, an intrinsic component of Cajal bodies, has no effect on Cajal bodies formation, but disrupts telomerase-telomere association, and abrogates telomere synthesis by telomerase (32). Similarly, hnRNP A2*, a slicing variant of hnRNP A2, interacts with hTR in Cajal bodies and plays a role in regulating telomerase recruitment to telomere and telomere elongation (38). With increasing number of newly identified Cajal body proteins (39), it is conceivable to speculate that some of them may participate in telomerase biology.

Venteicher *et al.* reported that knockdown of TCAB1 significantly reduced the number of detectable hTR foci and was associated with progressive telomere shortening (17). Moreover, telomere extension was inhibited by overexpression of a CAB box-mutant hTR that fails to accumulate in Cajal bodies (40), and Zhao *et al.* showed that long-term treatment with telomerase inhibitor, GRN163L, depleted hTR foci on telomeres and inhibited processive extension of telomeric DNA by telomerase (33). Based on these data and others, it has been proposed that Cajal body-associated hTR foci play a direct role in delivering telomerase to telomeres during S phase (21,35,40). In contrast, the present study demonstrates that in human cells hTR foci form in a Cajal body-independent but cell cycle-dependent manner (Figure 6B); therefore, we conclude that the capacity to form hTR foci is intrinsic, but it is only activated during S phase by factors yet to be identified. Similarly, we show that a subset of hTR foci colocalizes with telomeres in coilin-KO HeLa cells, indicating that telomerase trafficking and recruitment to telomeres is also Cajal body-independent. As mentioned above, it remains possible that non-coilin components of Cajal bodies, such as TCAB1, associate with hTR and/or hTERT, mediating transport, recruitment or localization of telomerase to telomeres in a cell cycle-dependent manner. Data to support this role for TCAB1 have been reported previously (19). In addition, data presented here strongly support a role for TPP1 in recruiting telomerase to the telomere during S phase, as suggested previously (36). Our previous work showed that multiple telomerases are pre-positioned to telomeres during S phase (33). These pre-positioned telomerases may correspond to TCAB1/hTR foci observed during late S and G2 in this study (Figure 6). TPP1 may be implicated in this process. The mechanism by which TPP1 recruits telomerase is not yet fully understood; however, it is likely that the OB-fold of TPP1 interacts with the hTERT-TEN CTE domain that may be required for telomerase aggregation (36).

In summary, this study provides new insight into telomere/telomerase biology in human cells, while raising questions about a previously proposed role for Cajal bodies in the maturation, assembly and trafficking of telomerase holoenzyme. Additional studies are needed to resolve these new questions and to identify specific roles that may exist for non-coilin components of Cajal bodies. Although KO of human coilin confers no major cellular phenotype upon HeLa cells, coilin-KO in mice is an embryonic lethal mutation. Thus, the exact biological and possibly species-specific biological role of coilin (at the level of the organism) remains poorly understood and is worthy of additional study.

## SUPPLEMENTARY DATA

Supplementary Data are available at NAR Online.

SUPPLEMENTARY DATA
