# Supplementary material for: Human cells lacking coilin and Cajal bodies are proficient in telomerase assembly, trafficking and telomere maintenance
Source: Nucleic Acids Res. 2014 Dec 3;43(1):385–95. doi: 10.1093/nar/gku1277 (PMC4288172; doi:10.1093/nar/gku1277)
Supplement: SUPPLEMENTARY DATA [file supp_43_1_385__index.html]

Human cells lacking coilin and Cajal bodies are proficient in telomerase assembly, trafficking and telomere maintenance — Human cells lacking coilin and Cajal bodies are proficient in telomerase assembly, trafficking and telomere maintenance — Human cells lacking coilin and Cajal bodies are proficient in telomerase assembly, trafficking and telomere maintenance — SUPPLEMENTARY DATA 

# Human cells lacking coilin and Cajal bodies are proficient in telomerase assembly, trafficking and telomere maintenance

## SUPPLEMENTARY DATA

**Files in this Data Supplement:**

- SUPPLEMENTARY DATA
