# Supplementary material for: Human cells lacking coilin and Cajal bodies are proficient in telomerase assembly, trafficking and telomere maintenance
Source: Nucleic Acids Res. 2014 Dec 3;43(1):385–95. doi: 10.1093/nar/gku1277 (PMC4288172; doi:10.1093/nar/gku1277)
Supplement: SUPPLEMENTARY DATA [file supp_gku1277_nar-02468-a-2014-File003.ppt]

## Slide 1
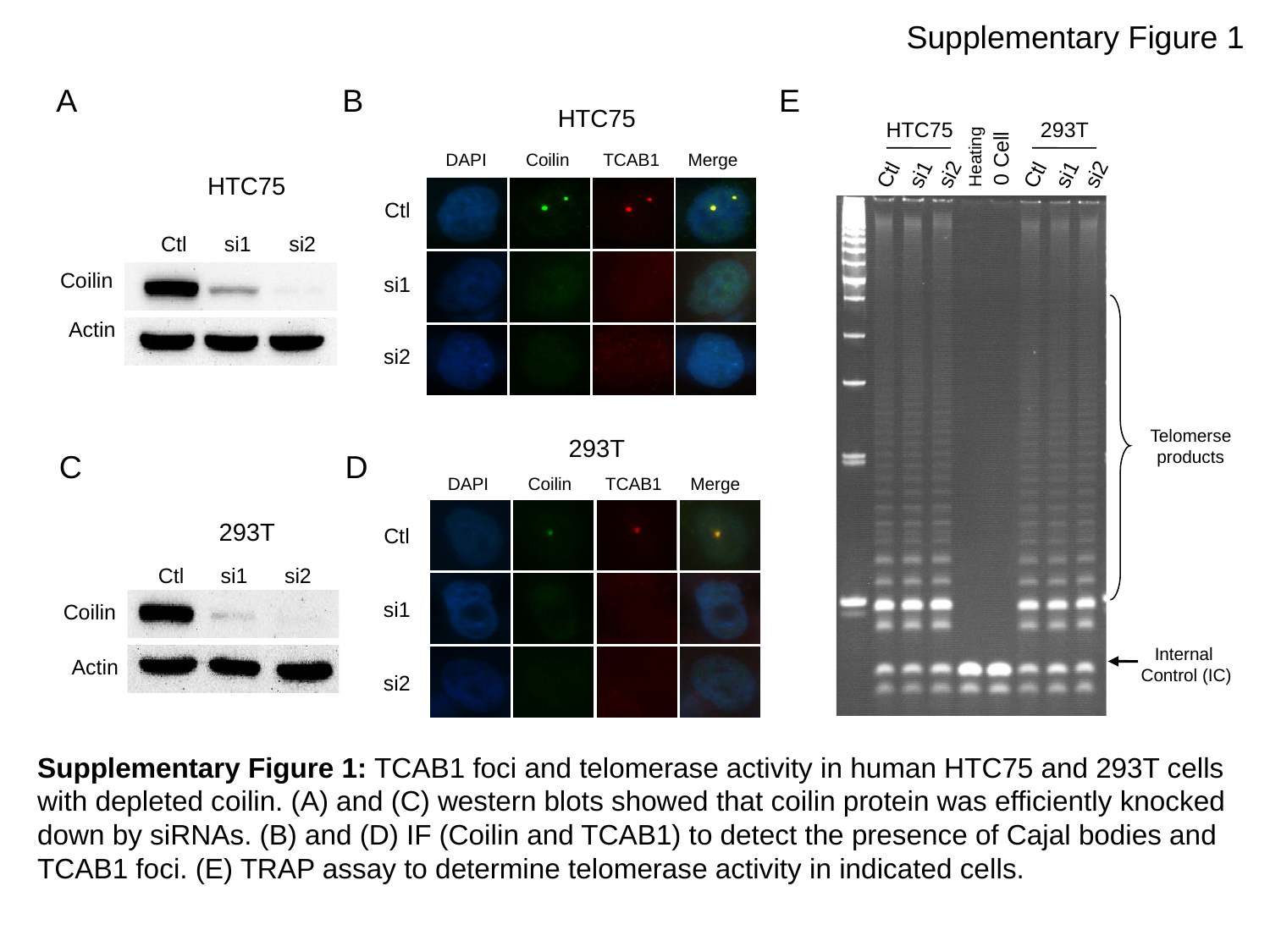

Supplementary Figure 1
A
B
E
HTC75
HTC75
293T
0 Cell
Heating
DAPI
Coilin
TCAB1
Merge
Ctl
si1
si2
si1
si2
si1
si2
Ctl
Ctl
HTC75
Ctl
si1
si2
Coilin
Actin
Telomerse
products
293T
C
D
DAPI
Coilin
TCAB1
Merge
Ctl
si1
si2
293T
Ctl
si1
si2
Coilin
Internal
Control (IC)
Actin
Supplementary Figure 1: TCAB1 foci and telomerase activity in human HTC75 and 293T cells with depleted coilin. (A) and (C) western blots showed that coilin protein was efficiently knocked down by siRNAs. (B) and (D) IF (Coilin and TCAB1) to detect the presence of Cajal bodies and TCAB1 foci. (E) TRAP assay to determine telomerase activity in indicated cells.

## Slide 2
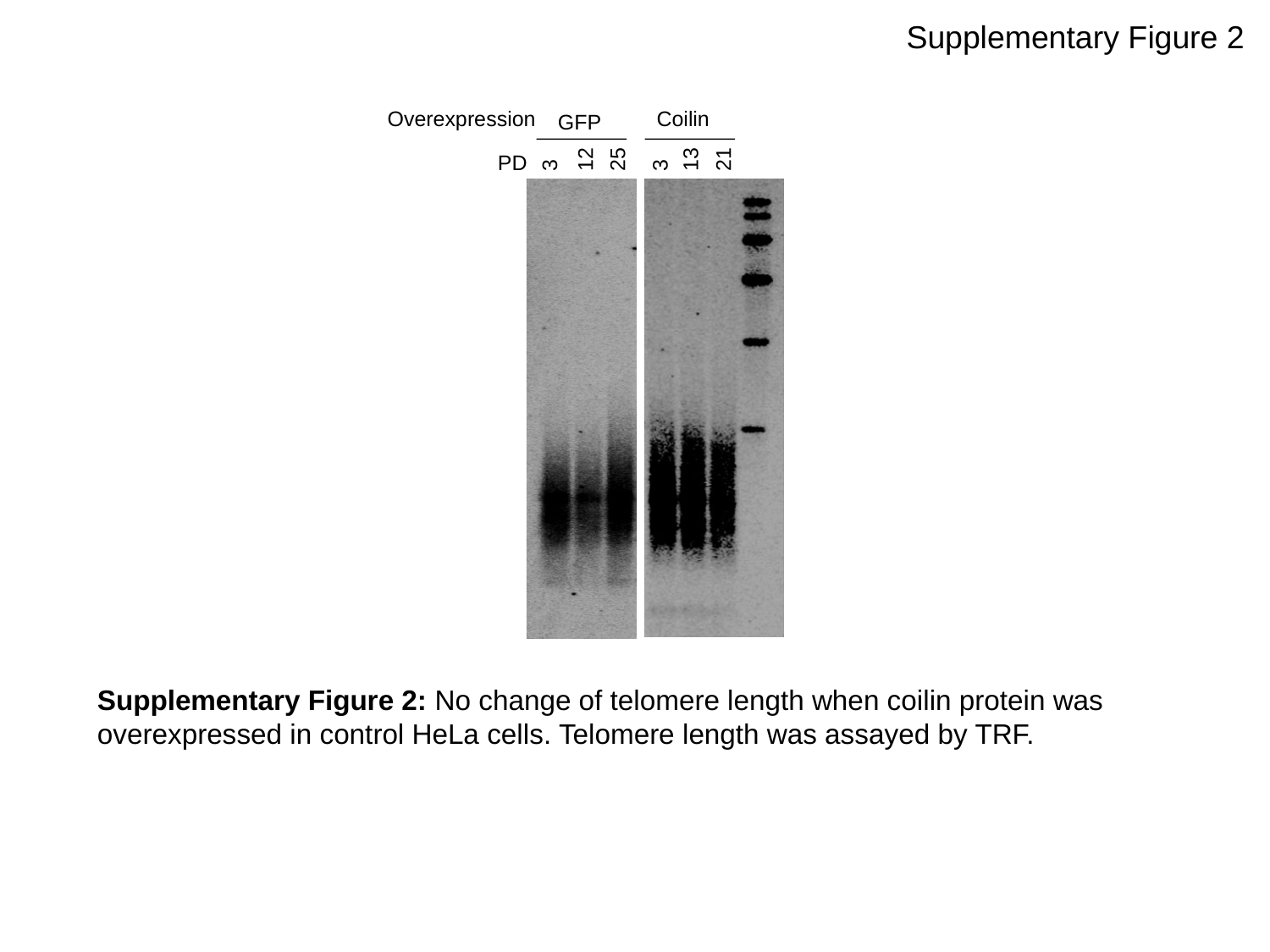

Supplementary Figure 2
Overexpression
Coilin
GFP
25
13
21
12
PD
3
3
Supplementary Figure 2: No change of telomere length when coilin protein was overexpressed in control HeLa cells. Telomere length was assayed by TRF.

## Slide 3
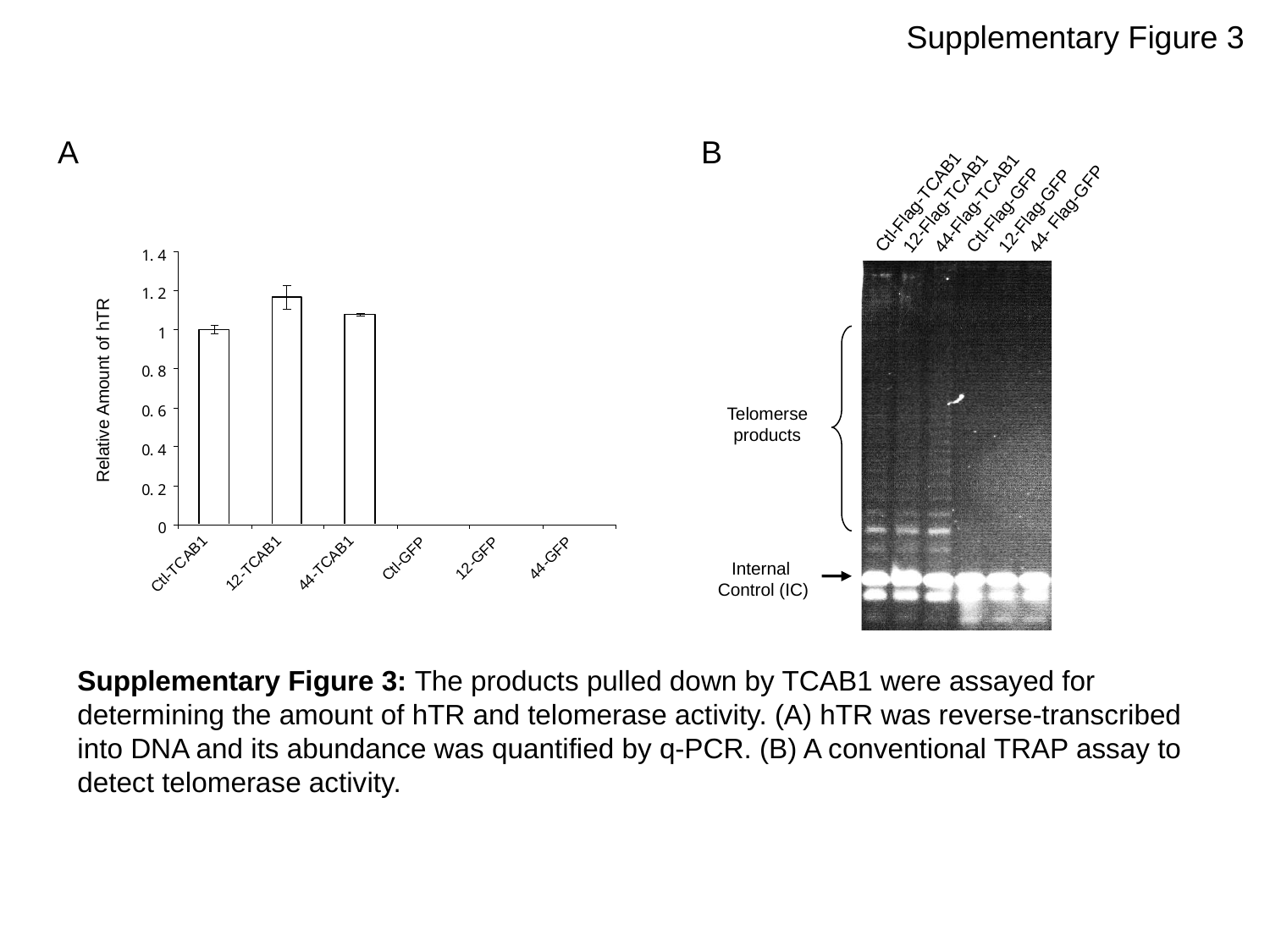

Supplementary Figure 3
A
B
Ctl-Flag-TCAB1
12-Flag-TCAB1
44-Flag-TCAB1
44- Flag-GFP
Ctl-Flag-GFP
12-Flag-GFP
Relative Amount of hTR
Telomerse
products
Internal
Control (IC)
Supplementary Figure 3: The products pulled down by TCAB1 were assayed for determining the amount of hTR and telomerase activity. (A) hTR was reverse-transcribed into DNA and its abundance was quantified by q-PCR. (B) A conventional TRAP assay to detect telomerase activity.

## Slide 4
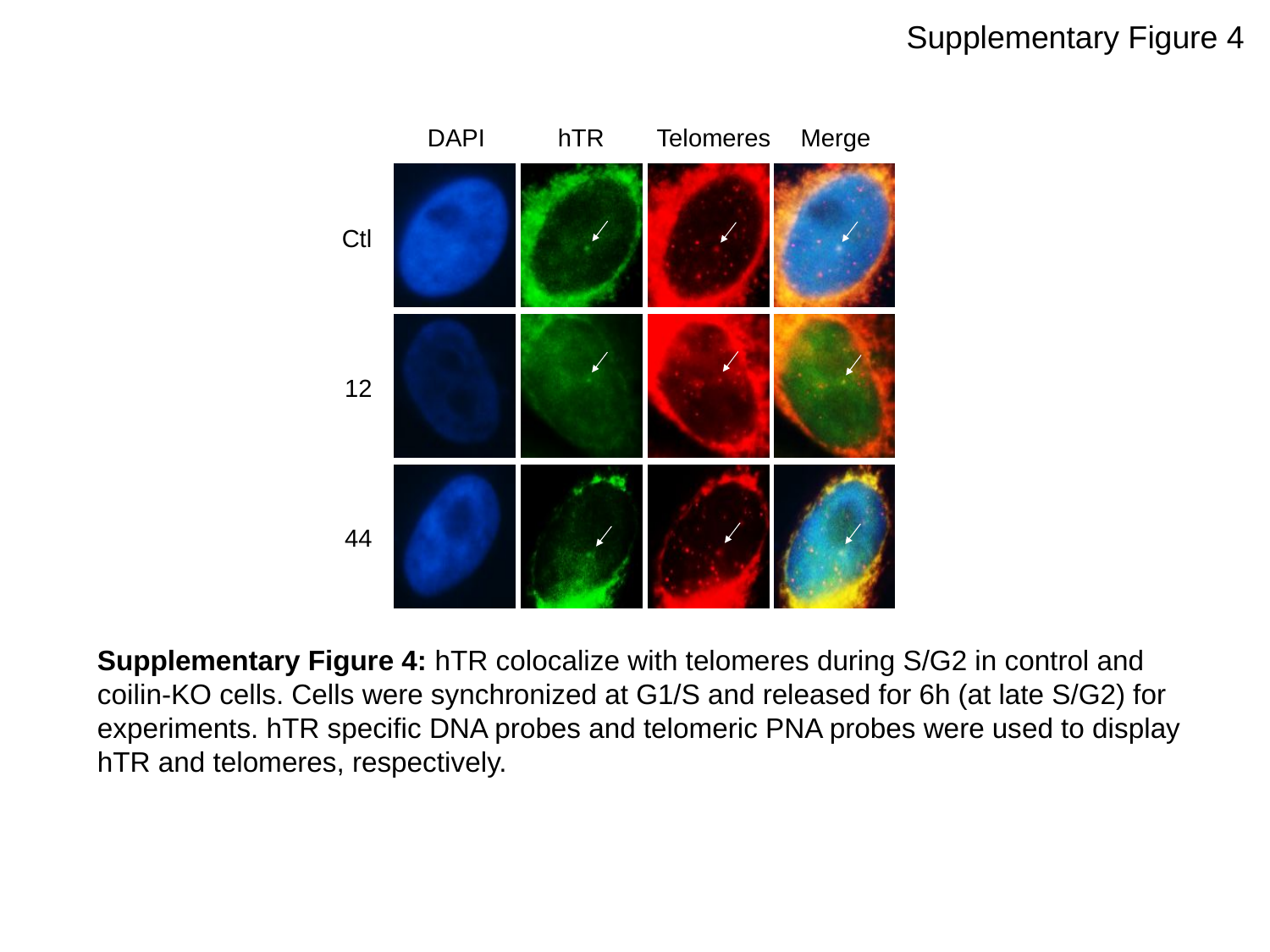

Supplementary Figure 4
DAPI
hTR
Telomeres
Merge
Ctl
12
44
Supplementary Figure 4: hTR colocalize with telomeres during S/G2 in control and coilin-KO cells. Cells were synchronized at G1/S and released for 6h (at late S/G2) for experiments. hTR specific DNA probes and telomeric PNA probes were used to display hTR and telomeres, respectively.
